# Sounds and beyond: multisensory and other non-auditory signals in the inferior colliculus

**DOI:** 10.3389/fncir.2012.00096

**Published:** 2012-12-11

**Authors:** Kurtis G. Gruters, Jennifer M. Groh

**Affiliations:** ^1^Department of Psychology and Neuroscience, Duke UniversityDurham, NC, USA; ^2^Department of Neurobiology, Duke UniversityDurham, NC, USA; ^3^Duke Institute for Brain Sciences, Duke UniversityDurham, NC, USA; ^4^Center for Cognitive Neuroscience, Duke UniversityDurham, NC, USA

**Keywords:** multisensory, inferior colliculus, auditory, sound localization, communication

## Abstract

The inferior colliculus (IC) is a major processing center situated mid-way along both the ascending and descending auditory pathways of the brain stem. Although it is fundamentally an auditory area, the IC also receives anatomical input from non-auditory sources. Neurophysiological studies corroborate that non-auditory stimuli can modulate auditory processing in the IC and even elicit responses independent of coincident auditory stimulation. In this article, we review anatomical and physiological evidence for multisensory and other non-auditory processing in the IC. Specifically, the contributions of signals related to vision, eye movements and position, somatosensation, and behavioral context to neural activity in the IC will be described. These signals are potentially important for localizing sound sources, attending to salient stimuli, distinguishing environmental from self-generated sounds, and perceiving and generating communication sounds. They suggest that the IC should be thought of as a node in a highly interconnected sensory, motor, and cognitive network dedicated to synthesizing a higher-order auditory percept rather than simply reporting patterns of air pressure detected by the cochlea. We highlight some of the potential pitfalls that can arise from experimental manipulations that may disrupt the normal function of this network, such as the use of anesthesia or the severing of connections from cortical structures that project to the IC. Finally, we note that the presence of these signals in the IC has implications for our understanding not just of the IC but also of the multitude of other regions within and beyond the auditory system that are dependent on signals that pass through the IC. Whatever the IC “hears” would seem to be passed both “upward” to thalamus and thence to auditory cortex and beyond, as well as “downward” via centrifugal connections to earlier areas of the auditory pathway such as the cochlear nucleus.

## Introduction

Organisms gather information about their environment from a variety of sensory systems, but how these sensory systems interact with each other is poorly understood. Multisensory integration is sufficiently common in cortical regions that the cortex has been described as a fundamentally multisensory processor [for review, see Ghazanfar and Schroeder (2006)]. In contrast, relatively little attention has been given to the subcortical systems that provide sensory information to the cortex. Much of the sensory information passed along to the cortex from subcortical areas is already multisensory in nature, and is influenced by behavioral state and relevance of stimuli. In this review, we focus on the multisensory and context-related connections and response properties of the inferior colliculus (IC), an important subcortical node in the auditory pathway.

The IC is particularly interesting in regard to multisensory and other non-auditory contributions to hearing as it is a necessary relay for nearly all ascending and descending auditory information [for review see Winer and Schreiner (2005)]. Situated relatively early in the auditory system, the IC is comprised of a central nucleus (ICC; see Table 1 for list of abbreviations) and various surrounding shell nuclei (shell nuclei of the IC^1^; sIC collectively), including, but not limited to, the external IC (ICX), pericentral nucleus of the IC (ICP), dorsal and lateral cortices of the IC, and brachium of the IC. The ICC primarily sends ascending auditory information to the thalamus (e.g., Kudo and Niimi, 1980; Calford and Aitkin, 1983) which then proceeds toward the cortex. The sIC also send ascending projections to the thalamus (ICP and ICX projections: Kudo and Niimi, 1980; and Calford and Aitkin, 1983) as well as the superior colliculus (SC) (brachium of the IC, ICX: Van Buskirk, 1983; dorsomedial part of the IC, ICX: Druga and Syka, 1984; ICX: Zhang et al., 1987; rostal pole of the IC: Harting and Van Lieshout, 2000), and descending information back to the auditory brainstem (including the ICC) (Huffman and Henson, 1990).

**Table 1:** **List of anatomical abbreviations**.

**Abbreviation**	**Full name**
AN	Auditory nerve
BLA	Basal lateral amygdala
DCN	Dorsal cochlear nucleus
FNc	Fastigial nucleus of the cerebellum
GP_C_	Caudal portion of the globus pallidus
IC	Inferior colliculus
ICC	Central nucleus of the inferior colliculus
ICP	Pericentral nucleus of the inferior colliculus
ICX	External nucleus of the inferior colliculus
NAm	Nucleus ambiguus
nDC	Dorsal column nuclei
PAG	Periaquaductal gray
SC	Superior colliculus
sIC	Shell nuclei of the inferior colliculus
SN_l_	Substantia nigra *pars lateralis*
TN	Trigeminal nerve
TN_op_	Ophthlamic branch of the trigeminal nerve
TNG	Trigeminal nerve ganglion
VTA	Ventral tegmental area

**Figure 1:**
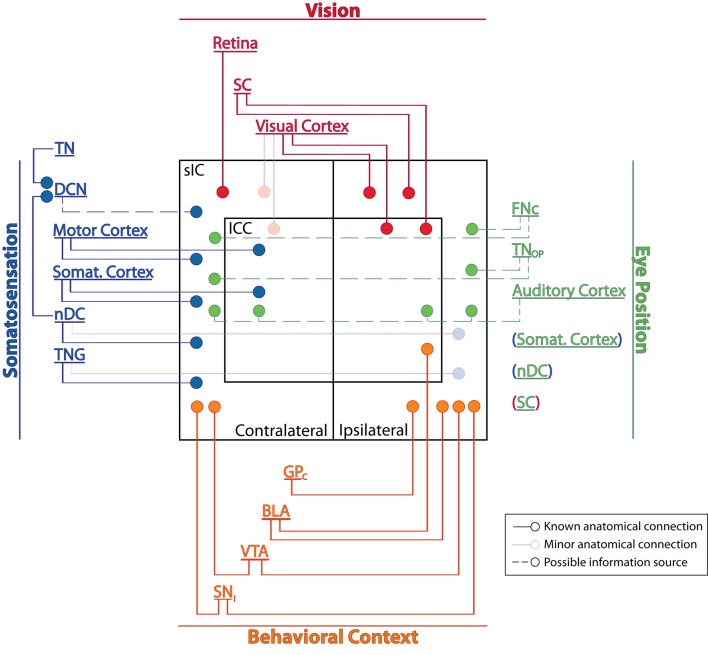
**Potential sources of non-auditory inputs to the IC.** Solid lines indicate probable primary sources of visual, somatosensory, or behaviorally relevant signals; these regions are known to process the corresponding type of information (e.g., the retina is involved in processing visual signals) and have direct connections to the IC. Faded lines represent sparse anatomical connections. Dashed lines are regions that, based on circumstantial evidence, may carry somatosensory or eye position signals to the IC. Somatosensory cortex, SC, and nDC may mediate more than one type of input and are listed under multiple headings; their connections are shown only once (indicated by parenthesis color). See Table 1 for abbreviations.

Converging anatomical and physiological evidence indicates that cells within the IC are sensitive to visual, oculomotor, eye position, and somatosensory information as well as to signals relating to behavioral context and reward (Figure 1). Auditory perception and behavior are likely to be shaped by these non-auditory inputs to the IC.

## Non-auditory influences on neural activity in IC

### Vision and oculomotor influences

Numerous anatomical studies have established the existence of direct retinal innervation of the contralateral IC (rat, monkey: Itaya and Van Hoesen, 1982; rat: Yamauchi and Yamadori, 1982; guinea pig, hamster, rat: Zhang, 1984; mole-lemming: Herbin et al., 1994). Projections from the retina pass through the contralateral SC and into the ICP near the midline, fanning out dorsolaterally, before being pruned back during early neural development (Cooper and Cowey, 1990). Enucleation of the contralateral eye causes the degeneration of these connections in adult animals (Paloff et al., 1985). In addition to receiving retinal efferents, both the ICC and sIC (in particular, the ICX) receive inputs from the visual cortex (primarily ipsilateral) (cat: Cooper and Young, 1976) and the ipsilateral SC, a visually responsive structure involved in programming saccadic eye movements (cat: Adams, 1980; bat: Covey et al., 1987; rat: Coleman and Clerici, 1987; barn owl: Hyde and Knudsen, 2000) [for review of saccade generation in the SC, see Gandhi and Katnani (2011)].

The presence of anatomical connections from visual processing sources suggests that IC neurons should be responsive to visual stimuli. Several physiological studies have investigated this hypothesis and established that there are cells in the IC whose auditory responses are modulated by a concurrent visual stimulus (Syka and Radil-Weiss, 1973; Tawil et al., 1983), or that are capable of responding directly to visual stimuli without an accompanying sound (Mascetti and Strozzi, 1988; Porter et al., 2007; Bulkin and Groh, 2012b). Early reports using anesthetized and paralyzed cats suggested that 8–9% of IC neurons were visually responsive (Tawil et al., 1983: 112 total cells tested; Mascetti and Strozzi, 1988: 91 total cells tested), but later work involving more extensive statistical testing as well as awake and behaving monkeys performing a visually guided saccade task suggests that the proportion is much higher (Porter et al., 2007; Bulkin and Groh, 2012b). Porter et al. (2007) found a variety of response profiles including excitation (35%) or inhibition (5.5%) in response to a visual stimulus, excitation in conjunction with a visual stimulus and its accompanying saccade (15%), or excitation just during the saccade (4.5%). An additional 6% exhibited delayed activity increases or activity that differed in a non-specific but statistically significant fashion from baseline during stimulus presentation and/or the saccade. Overall, 64% of the tested neuronal population (*n* = 180) displayed statistically significant responses to visual stimuli and/or saccade-related activity.

**Figure 2:**
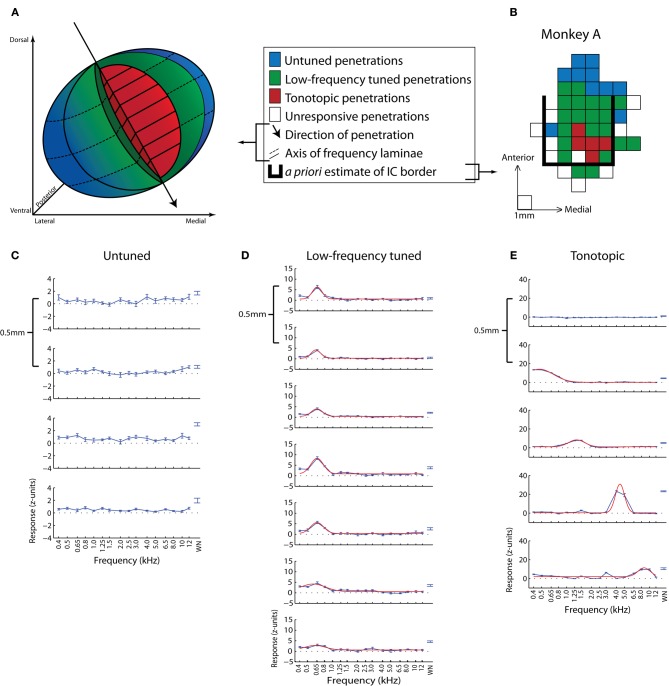
**Map of sound frequency representation in the monkey IC. (A,B)** Three-dimensional schematic of sound frequency representation **(A)** based on a representative example dataset **(B)** from Bulkin and Groh (2011). Untuned sites are presumed to be in the outer sIC, low-frequency tuned sites from near the sIC-ICC border, and tonotopic sites from the center of the ICC. The grid in **(B)** is a top-down view of the recording grid for one monkey in the study. Each square represents a penetration site and is colored based on the response properties of that site. The bold black line delineates the *a priori* estimated boundaries of the IC based on MRI scans. **(C–E)** Example penetrations from untuned **(C)**, low-frequency tuned but not tonotopic **(D)**, and tonotopic **(E)** penetration locations. Each series is comprised of multiple recordings taken from a single example penetration. Frequency response functions were recorded at 0.5 mm increments along the dorsolateral-ventromedial penetration axis. The first graph in each series corresponds to the first, i.e., most dorsal, site to show responses to auditory stimuli along that penetration. Absolute depths of first responsive sites varied across penetrations. Blue lines **(C–E)** indicate the normalized average response over a 200 ms period as a function of sound frequency, and red lines **(D,E)** show the best Gaussian curve fit to tonal data. **(B–E)** Adapted from Bulkin and Groh (2011).

A more detailed mapping of the locations of visual- and saccade-related responses in the IC has revealed that visual response properties are not uniform throughout the IC. Bulkin and Groh (2012b) localized visually responsive neurons with respect to a previous systematic mapping of auditory function in the region (Bulkin and Groh, 2011) (Figure 2). To define this functional map, Bulkin and Groh (2011) used a combination of electrophysiological recording, stereotaxic coordinates, MRI, histology, and known physiological properties of IC subdivisions to identify and demarcate three regions with distinct response properties. A central tonotopic region (red region in Figures 2A,B; example penetration given in Figure 2E), likely situated well within the ICC, exhibited cells whose best frequency increased as the electrode advanced along a dorsolateral to ventromedial trajectory. The surrounding region contained neurons tuned for low-frequency sounds but lacked a tonotopic gradient (low-frequency tuned area; purportedly overlapping with the outer portions of the ICC as well as the sIC) (green region of Figures 2A,B; example penetration in Figure 2D). Cells within the final region at the periphery of the IC were either unresponsive to pure tones or non-selective for tone frequency (blue region of Figures 2A,B; example penetration in Figure 2C).

Visual- and visuomotor-responses were found distributed throughout the IC (Bulkin and Groh, 2012b). However the different regions exhibited different proportions of sites sensitive to visual stimuli as well as different patterns of responses at those sites. Untuned auditory areas showed robust visual- or saccade-related responses in 81% of the tested units. Low-frequency tuned sites also showed vigorous visual- and saccade-related firing, but the responses were less strong than untuned sites and were found in only 31% of these cells. Tonotopic penetrations yielded the lowest proportion of responsive cells (26%). Visual-related responses observed on these penetrations were weak, deviating only slightly from baseline, and did not exhibit the same clear stimulus- or saccade-onset timing present in untuned and low-frequency tuned populations.

The relative contributions of each of the potential anatomical sources of visual input to the IC are not known. Response latency is only moderately informative: signals within the IC cannot occur faster than their source signal, but reported latencies span a range consistent with any of the potential sources. Tawil et al. (1983) reported latencies ranging between 20 and 30 ms for the nine visually responsive cells recorded from within the cat IC, consistent with direct retinal innervation (latency in cat optic tract ≈20 ms: Freund et al., 1972), but excluding presumably longer latency inputs from the SC or visual cortex. However, in monkeys, latencies were reported to range from 60 to 115 ms (Porter et al., 2007), compatible with visual latencies from visual cortex, SC, and even the retina under low intensity stimulation (latency in monkey visual cortex ≈55–130 ms: Schmolesky et al., 1998; monkey SC ≈ 60–100 ms: Bell et al., 2006; monkey optic tract ≥40 ms: Inoue et al., 2000).

Likewise, the properties of the visual receptive fields (vRFs) found in the IC do not obviously implicate any one of the known inputs to the exclusion of the others. Like all three potential input sources, the IC's representation favors the contralateral field, but unlike the possible inputs, no topographic organization within the contralateral field has been observed in mammals. The vRFs of individual cells are variable in size, ranging from tens of degrees to nearly half of the visual field (Mascetti and Strozzi, 1988; Porter et al., 2007; Bulkin and Groh, 2012b). This is larger than the typical RFs found in retinal ganglion cells, visual cortex, or the SC (striate cortex: Wurtz, 1969; SC: Goldberg and Wurtz, 1972; retinal ganglion cells: Hammond, 1974; and Cleland et al., 1979).

The types of stimulus selectivity seen in IC neurons are also evident in more than one of the potential input sources. For example, visually responsive neurons in the IC tend to respond well to both static (Porter et al., 2007; Bulkin and Groh, 2012b) and moving (Mascetti and Strozzi, 1988) stimuli, properties observed in both the visual cortex (Wurtz, 1969) and retina (Shapley and Perry, 1986). IC cells that respond to moving stimuli are not tuned to a particular direction of stimulus motion (Mascetti and Strozzi, 1988), which is similar to a subset of SC neurons that are directionally untuned (Humphrey, 1968; Rhoades and Chalupa, 1976). Thus, the retina, visual cortex, and the SC all share some aspects of stimulus selectivity with IC neurons. Additional research is therefore necessary to explore receptive fields and stimulus selectivity in more detail before reaching conclusions on the potential source, or synthesis of multiple sources, responsible for IC vRFs and response properties.

**Figure 3:**
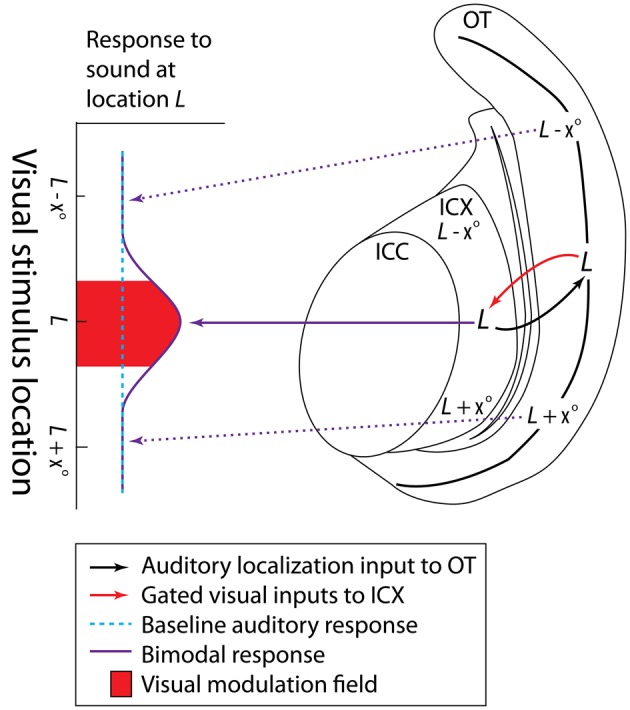
**Visual information modulates activity of cells in the owl ICX when auditory and visual inputs are closely aligned in space.** Cells in the owl ICX respond best to a sound from some fixed location *L* in space (determined by interaural timing and level cues) (dotted blue line; note that the location of the auditory stimulus does not change in this schematic) and that information is transmitted to the corresponding location within the OT (black arrow). When a visual cue occupies the same or some nearby location in space, gated feedback from the OT (red arrow) enhances auditory responses (solid purple arrow); visual cues at other locations will not enhance auditory responses (dotted purple arrows). The visual field corresponding to ≥50% of the max auditory modulation is defined as the visual modulation field (red field). Adapted from Gutfreund et al. (2002); and Bergan and Knudsen (2009).

Visual sensitivity has also been reported in the barn owl IC, but in contrast to mammalian studies, visual spatial sensitivity in the barn owl ICX appears to be well organized and tuned to the auditory space map in the same region (Bergan and Knudsen, 2009). Visual modulation fields [vMFs; defined as the region of visual space in which a stimulus exhibits ≥50% of its maximal modulatory effects on the paired auditory stimulus (see Figure 3)] of neurons throughout the ICX are well correlated with the location predicted by auditory localization cues. That is, a visual stimulus occurring at a given location in the visual field can modulate the ICX response to sounds originating from a similar point in space (Figure 3). Also in contrast to mammals, neurons in the owl ICX do not appear to respond to visual stimuli alone (Gutfreund et al., 2002; Bergan and Knudsen, 2009). Instead, visual information projecting from the owl's optic tectum (OT; homologous to the mammalian SC) to the ICX is gated via GABAergic inhibition in the OT (Gutfreund et al., 2002). Multiunit activity in the ICX is sensitive to visual stimulation only if inhibition is blocked in the region of OT corresponding to those units' preferred auditory location.

A variety of potential factors might influence the proportions and response patterns of visually sensitive cells in the IC. First, different species may utilize visual information in different ways depending on their ecological niche. For instance, diurnal species such as monkeys (e.g., Porter et al., 2007; Bulkin and Groh, 2012b) may exhibit response patterns that differ from those of nocturnal species such as cats or barn owls (e.g., Tawil et al., 1983; Mascetti and Strozzi, 1988; Bergan and Knudsen, 2009). Second, the cognitive state of the animal may be an important factor: Porter et al. (2007), and Bulkin and Groh (2012b) both used awake and behaving animals and found the highest proportion of visually responsive cells in comparison to other studies. Bergan and Knudsen (2009) estimated a slightly lower proportion in their awake but restrained birds^2^, while the anesthetized preparations used by Tawil et al. (1983) and Mascetti and Strozzi (1988) yielded the smallest proportions. As discussed in detail in the section on “Behavioral context” of this review, behavioral state has a profound effect on neural activity in the IC. The report by Gutfreund et al. (2002) suggests that behavioral state might exert a specific influence over visual responsiveness in the IC by gating the passage of visual information into the IC. Importantly, the SC (or OT) is commonly implicated in both oculomotor (Gandhi and Sparks, 2007) and attentional control (e.g., Shen et al., 2011), and serves as one of the routes by which visual input can reach the IC. The use of a saccade task might therefore have been an important factor increasing the proportion of visually responsive cells in both Porter et al. (2007) and Bulkin and Groh (2012b). Other influential factors may include the breadth of sampling of visual space and the type of visual stimuli used. Visually responsive neurons in other brain areas are known to respond only to stimuli presented within spatially restricted receptive fields and to stimuli of preferred orientations, colors, shapes, and so forth (Schiller, 1986). It is likely that the set of visual stimuli used in the aforementioned studies did not exhaust all of the parameters for which visually responsive cells in the IC are selective. Therefore, the reported proportions may well be an underestimate of the actual proportion of visually responsive cells.

The functional purpose of visual signals in the IC is unknown. One potential role is to help calibrate the representation of auditory space. This hypothesis has been tested thoroughly in the owl [for a review, see Knudsen (2002)], where visual space maps in the OT are functionally and anatomically connected to the auditory space maps of the ICX (Bergan and Knudsen, 2009). Barn owls reared with prisms that displace visual space show altered auditory spatial sensitivity in the ICX (Brainard and Knudsen, 1993), altered connectivity between the ICC and ICX (Debello et al., 2001), and altered connectivity patterns from the ICX to OT (Hyde and Knudsen, 2002; Linkenhoker and Knudsen, 2002). Ocular enucleation in rats appears to alter auditory spatial sensitivity as well (Pageau et al., 2008). This may be due to visual deprivation, though impaired eye position signals are also likely to result from enucleation and should not be dismissed as a contributing factor (see the section on “Eye position”). Together, these studies indicate that coding of auditory location is at least partially dependent on visual input. However, calibration of auditory space may not be the only role of visual signals in the IC. A possible role of visual signals in the sIC for communication is discussed in the section on “Communication.”

### Eye position

**Figure 4:**
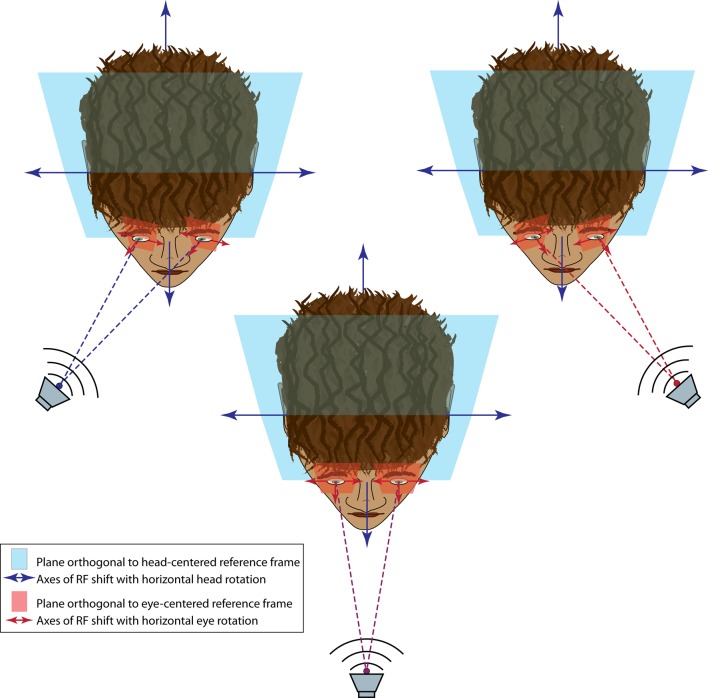
**Audiovisual reference frame problem.** When an audiovisual target is straight ahead (center panel), the auditory and visual reference frames coincide; that is, a plane orthogonal to the auditory head-centered reference frame (blue field) is parallel to the plane orthogonal to the visual eye-centered reference frame (red field), and both are perpendicular to the audiovisual information source (speaker). When the target is moved eccentrically (upper panels), the head-centered and eye-centered reference frames are no longer parallel, with the eye-centered reference frame having rotated around a vertical axis (for horizontal eye movements). In this example, the eye-centered reference frame is still perpendicular to the audiovisual target but the head-centered reference frame is not.

The eyes and ears necessarily receive visual and auditory spatial information, respectively, within a different frame of reference. Specifically, visual space is initially encoded based on where the image falls on the retina (a so-called eye-centered reference frame) while auditory space is calculated based on the position of the sound relative to the ears and the head (a head-centered reference frame). In species where the eyes are able to move to a substantial degree within the head (e.g., rhesus monkeys and cats but not rodents or barn owls), the visual and auditory reference frames are not fixed to each other (Figure 4). Because of this constantly changing relationship between reference frames, aligning visual and auditory space requires factoring in both the orbital position of the eyes and sound localization cues.

**Figure 5:**
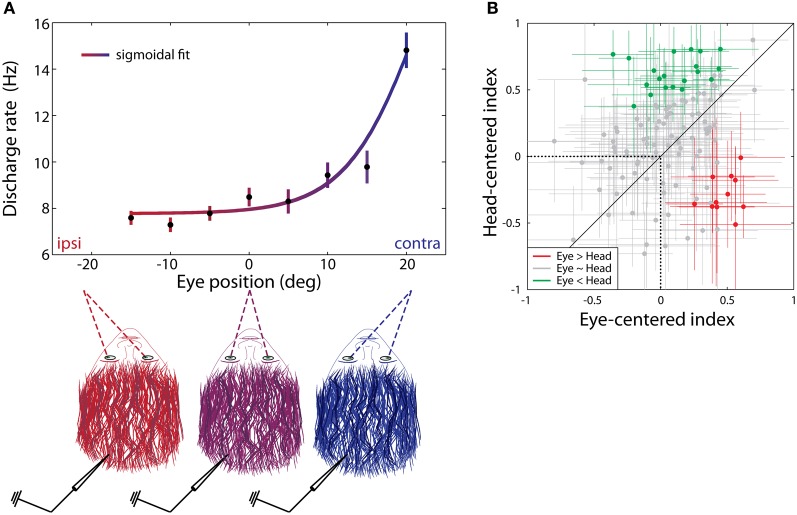
**Eye position gain and reference frame of IC cells. (A)** Contralateral gain as a function of eye position in azimuth. The firing rate of eye position sensitive cells in the IC tends to increase with contralateral fixations. **(B)** The reference frame of individual IC cells was determined by calculating correlation coefficients between eye position during sound presentation, and sound locations defined (1) with respect to the eyes (eye-centered index) and (2) with respect to the head (head-centered index). A value of 1 indicates that the response function of a cell is perfectly aligned with the sound source in a particular reference frame (eye-centered or head-centered), while a value of −1 indicates perfect anti-correlation. Crosses centered on individual points indicate 95% confidence intervals for reference frame values. The reference frame of individual cells may be more head-centered (green) or eye-centered (red), or cells may have a hybrid reference frame (gray). **(B)** Reprinted from Porter et al. (2006).

Groh et al. (2001) identified a subset of cells in the IC (approximately one-third: 24/73) that altered their firing patterns in response to an auditory stimulus depending on where the eyes were positioned in their orbits with respect to the head. More specifically, the firing rate of individual neurons usually tended to increase as a monotonic function of eye position, with the population favoring contralateral eye positions (relative to IC hemisphere) (Porter et al., 2006) (Figure 5A). Additionally, eye position sensitivity appeared to be more common among neurons insensitive to sound location (up to 32% for sound location sensitive units and 56% for insensitive units; Porter et al., 2006), suggesting somewhat segregated subpopulations for eye position and sound location sensitive units in the IC. Eye position sensitivity has also been found for vertical eye positions (Zwiers et al., 2004; Bulkin and Groh, 2012a), as well as during both task-related and spontaneous fixations, and with or without the presence of a concurrent sound stimulus (Porter et al., 2006; Bulkin and Groh, 2012a). Collectively, these results indicate that eye position signals are an important aspect of IC processing and are separable from signals related to head-centered sound location, vision, and eye movements.

The reference frame in the IC has been found to be neither head-centered nor eye-centered but somewhere in between. Porter et al. (2006) reported that only 16% of tested units were head-centered, whereas 9% were more eye-centered; the remaining 75% of the tested units were approximately equally consistent with either head- or eye-centered reference frames (Figure 5B). A model trained on these data was able to provide an output that closely approximated a particular sound location via either head- or eye-centered coordinates (Porter et al., 2006). The brain seems to maintain this hybrid encoding scheme until the time of saccade generation. Specifically, the hybrid scheme is similar in the IC, auditory cortex (Werner-Reiss et al., 2003; Porter et al., 2006), and the visual intraparietal sulcus (Mullette-Gillman et al., 2005, 2009) as well as in the sensory signals in the SC (Jay and Sparks, 1984; Populin et al., 2004; Lee and Groh, 2012). However, a motor output command is eventually developed in eye-centered coordinates in the SC at the time of saccade generation (Lee and Groh, 2012).

The location of eye position sensitive cells within the IC was recently mapped in detail (Bulkin and Groh, 2012a) with respect to the functional response map previously described (Bulkin and Groh, 2011; see the section on “Vision and oculomotor influences,” and Figure 2). Eye position sensitivity was found throughout the IC, but the proportions varied according to the functional response patterns of the recording site. Eye position effects were detected in 25% of tonotopic sites, 33% of low-frequency tuned sites, and 42% of untuned sites. As noted previously, these responses are likely to coincide with the central-most part of the ICC, the edges of the ICC and the inner part of the sIC, and the outer sIC respectively (Bulkin and Groh, 2011).

Despite the physiological studies identifying eye position signals in the IC, the anatomical sources of these signals have yet to be identified. It is not clear whether such signals are a result of corollary discharge from oculomotor outputs [e.g., the SC, or the fastigial nucleus of the cerebellum (Carpenter, 1959; Earle and Matzke, 1974)], proprioceptive feedback from muscles controlling eye position [along, for instance, the opthalamic tract of the trigeminal nerve (TN) (Steinbach, 1987); cuneate nuclei (Porter, 1986); or from the somatosensory cortex (Zhang et al., 2008)], or some combination of both. These signals may also come from other auditory areas. For instance, the primary auditory cortex contains eye position signals (Werner-Reiss et al., 2003) and sends anatomical projections to the IC (Winer et al., 1998). It is possible that eye position signals are sent through this route via corollary discharge from cortical regions important for eye position and movements (after Sommer and Wurtz, 2008). Further investigation will be necessary to identify the connectivity of eye position sensitive neurons in the IC.

### Somatosensation

The IC also receives input from the somatosensory system. Connections from various brainstem nuclei—including the trigeminal nerve ganglion (TNG) and nuclei of the dorsal column (nDC)—have been identified in both the intercollicular region (cat: Anderson and Berry, 1959; opossum: Robards et al., 1976), and the ICX (opossum: Robards, 1979; cat: Aitkin et al., 1981; monkey: Wiberg et al., 1987; rat: Coleman and Clerici, 1987; hedgehog: Kunzle, 1998). Additionally, both the motor and somatosensory cortices project to the ipsilateral ICC and ICX (Cooper and Young, 1976). The ICC may also receive somatosensory signals from other areas along the auditory pathway. For example, the ICC is heavily innervated by the dorsal cochlear nucleus (DCN) (e.g., Cant and Benson, 2008), which is also known to receive direct innervation from the nDC (Li and Mizuno, 1997) and the TN (Zhou et al., 2007), and whose principle output cells are modulated by TN stimulation (Koehler et al., 2011). The ICX, meanwhile, receives convergent inputs from the DCN and spinal trigeminal nucleus (Zhou and Shore, 2006), suggesting that multiple, overlapping circuits may be involved in creating somatosensory-sensitive cells in the IC.

As suggested by these anatomical connections, neurons in both the ICC and ICX are sensitive to somatosensory inputs from the spinal cord [i.e., inputs from the spinal dorsal column (DC), peripheral nerves, and body, presumably via the nDC]. Specifically, cells may respond to unimodal somatosensory stimulation or alter their firing rate to auditory stimuli when sounds are paired with concurrent somatosensory stimulation. This was first demonstrated in the IC of anesthetized rats, where neurons changed their response to a pure tone stimulus in the presence of concurrent sciatic nerve stimulation (Syka and Radil-Weiss, 1973). Similar results have been reported using electrical stimulation of the median nerve in anesthetized rats (Szczepaniak and Moller, 1993) as well as the tibial nerve (Aitkin et al., 1978) and DC (Aitkin et al., 1978; Tawil et al., 1983) of anesthetized cats. In the case of DC stimulation, ≈5–20% of the IC cell population responded to electrical DC stimulation alone (this range may be partially due to variations in stimulation parameters across studies) and roughly 55% responded differentially to a combination of DC stimulation and concurrent sound presentation compared to sound alone. DC stimulation enhanced the acoustic response in about one-third of the bisensory units and inhibited the other two thirds. Additionally, cells in the ICX of anesthetized cats were found to respond to manual tactile stimulation of the skin and hair across the entire surface of the body (individual neurons had bodily receptive fields at varying locations and of variable sizes, with the full body surface represented across the population) (Aitkin et al., 1978, 1981). The proportion of cells responsive to tactile stimulation was somewhat lower than that responsive to DC stimulation: out of 261 cells (Aitkin et al., 1981) only 16% responded to unimodal tactile stimuli while 4% responded differentially to concurrent tactile and auditory stimulation compared to sound alone.

In addition to inputs carried via the spinal cord, the IC is also sensitive to influences mediated via cranial nerves, specifically from the TN and TNG. Electrical stimulation of the TNG in the absence of a sound stimulus causes increased metabolic activity within the IC cell population when compared to non-stimulated control animals [as measured by uptake of [14C]2-deoxyglucose (2DG); El-Kashlan and Shore, 2004]. Moreover, uptake of 2DG in response to TNG stimulation was qualitatively similar in the IC to uptake in response to sound stimulation. Similarly, electrical TN stimulation paired with sound modulated the response patterns of approximately two-thirds of the cells in the ICX of anesthetized guinea pigs (Jain and Shore, 2006). Specifically, auditory responses were inhibited by paired TN stimulation in nearly half of the tested units (60/126, 48%) and enhanced in 23 units (18%). TN stimulation alone did not appear to elicit responses in the absence of a sound.

Functionally, somatosensory inputs to the IC may serve numerous different roles within the auditory system^3^. Consider, for instance, the proposed role for eye position signals within the IC: aligning the neural representation of visual and auditory reference frames. In a relatively simple case of fixating on a sound source near the fovea, eye position and sound location cues appear to be sufficient information to execute the appropriate saccade. In more complex cases involving head, trunk, and limb motion, the brain must coordinate numerous effector muscles in order to orient toward (or away from) a sound source. Eye position, and possibly somatosensory, signals in the IC likely provide the information necessary to localize and act on some sound source. Additionally, the orientation of the pinnae must be factored into the interpretation of direction-dependent spectral cues in species that make guided ear movements in response to sounds (e.g., the cat: Populin and Yin, 1998). This information appears to be present in the DCN via nDC and TN inputs (Kanold and Young, 2001), and may be transmitted to the IC either directly from one or both of these somatosensory nuclei, or by way of the DCN. The IC, therefore, is presumably important for this behavior in that it may contribute sound location cues relative to eye position and pinnae-position to higher-order orientation circuitry.

Moreover, reflexive auditory orientation and startle behaviors involving the head may involve IC signaling (e.g., Leitner and Cohen, 1985). Thompson and Masterton (1978) found that shallow lesions at the level of the IC degrade the accuracy and latency of reflexive head orientation toward unexpected sounds in cats, but the response was still initiated toward the correct hemifield. In contrast, deep lesions that sever the connections from the DCN to IC cause startle responses targeted toward the wrong hemisphere. Such behaviors may be different from saccades: the reaction times (40 ms on average) were much faster than typical saccade latencies (150–300 ms: e.g., Carpenter, 1988; Jay and Sparks, 1990). These data suggest that an auditory orientation reflex is dependent on the IC and its inputs. Reflexive orientation likely requires a body-to-head (or head-to-body) reference frame transformation to execute a response in the appropriate direction, and the convergence of somatosensory and sound location information in the IC indicates that this is possible.

In addition to contributing to these various types of sound localization behaviors, all of these input sources would be useful in suppressing self-generated noise, including vocalizations, mastication, and respiration (Jain and Shore, 2006), as well as locomotion and visceral function. Motor structures may send corollary discharge signals to the IC (and other auditory regions) to suppress noises resulting from the ensuing behaviors. An example of this has been observed in crickets: auditory processing regions are inhibited via corollary discharge of the motor signals used to produce singing behaviors (Poulet and Hedwig, 2002). Similar mechanisms for attenuating self-generated sounds have been observed in more complex neural systems, including the bat auditory system (Suga and Shimozawa, 1974).

### Behavioral context

**Figure 6:**
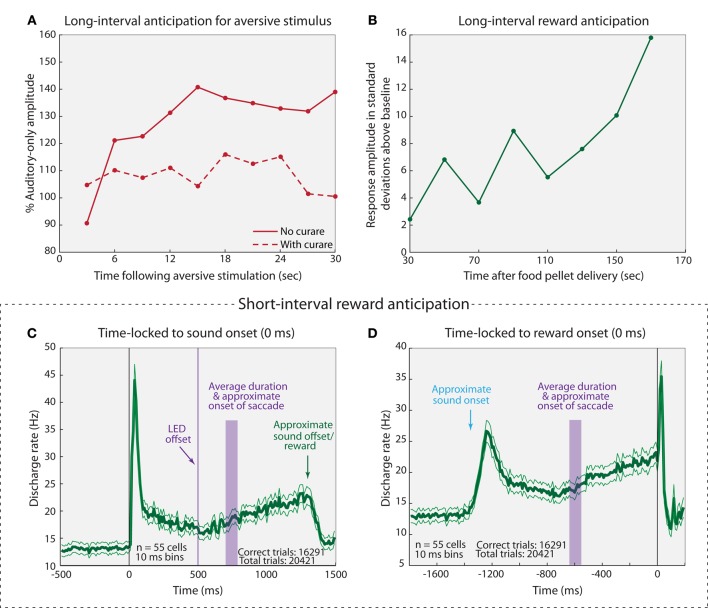
**Anticipatory responses of IC cells. (A)** Average-evoked potential recorded from the IC in response to sounds presented at 3 s intervals following aversive stimulation of the mesencephalic central gray (MCG) with (dashed line) and without (solid line) curare. MCG stimulation occurred at a fixed interval of 30 s (thus, 0 s post-MCG stimulation may be thought of as 30 s pre-MCG stimulation for the following trial). **(B)** Representative example of a multi-unit response to sound presentation following rewarding stimulus. Sounds were played at varying intervals (up to 180 s) following reward, and the next reward was delivered after a second variable interval. Note that no error bars were provided in the source materials for panels **(A,B)** so the variability and potential statistical significance of the finding cannot be assessed. **(C,D)** Average response (± standard error) of IC cell population time-locked to sound onset **(C)** and reward onset **(D)**. Briefly, monkeys fixated on an LED while a sound was played. The LED was extinguished and the monkeys made a saccade to the auditory target. They were rewarded if the auditory saccade was within the appropriate fixation window (8–11°, depending on target distance from LED). Both rewarded and unrewarded trials were included in this analysis. Adapted from **(A)** Ruth et al. (1974); **(B)** Nienhuis and Olds (1978); **(C,D)** Metzger et al. (2006).

An animal's behavioral state influences neural activity in the IC, seemingly depending on task engagement and expected outcome. In rats, the activity of cells in the IC was found to increase as an expected rewarding (Nienhuis and Olds, 1978) or aversive (Ruth et al., 1974) stimulus draws near, at reinforcement intervals ranging from seconds to minutes (Figures 6A,B). Curare blocks the apparent anticipatory build up response for aversive stimulation, indicating that acetylcholine plays a role in this build up activity (Ruth et al., 1974). Similar reward-anticipation and task performance effects have been observed in the monkey IC. An estimated 60% of IC neurons show a general increase in firing rate when a monkey is engaged in active behavior as opposed to passive listening (46/80 cells: Ryan and Miller, 1977; Ryan et al., 1984). Additionally, the activity of IC neurons increases in apparent anticipation of a reward (Figures 6C,D), and the amount of increase depends on the size of the reward (Metzger et al., 2006). The trend appears to be the same in humans: attending to changing pitches in one ear while ignoring pitch changes in the other activates the contralateral IC (relative to attended ear) more than the ipsilateral IC (Rinne et al., 2008). Collectively, these data indicate that cells within the IC are sensitive to engagement in a task, and that they exhibit anticipation for some upcoming rewarding or aversive stimulus. It is particularly striking that these cells seem to be capable of anticipating over extended time periods of 30 s or more. Presumably, the increased activity reflects heightened sensitivity to behaviorally relevant stimuli.

These findings are consistent with known anatomical connections to the IC from regions typically associated with subjective value and emotion. Specifically, the sIC receive inputs from the ipsilateral caudal globus pallidus (GP; for cats and rodents in which these tracing studies have been carried out, the GP is equivalent to the external capsule of the GP in primates) (inputs primarily target ICX) (Yasui et al., 1990; Shinonaga et al., 1992; Shammah-Lagnado et al., 1996) as well as GABAergic inputs from the substantia nigra pars lateralis, bilaterally (SN_1_) (specified ICX: Coleman and Clerici, 1987; unspecified: Yasui et al., 1991; and Moriizumi et al., 1992). The ICX also receives bilateral projections from the ventral tegmental area (VTA) (Herbert et al., 1997) while both the ICC and sIC receive ipsilateral inputs from the basal nucleus of the amygdala (Hopkins and Holstege, 1978; Marsh et al., 2002). All of these regions have a substantial body of literature implicating them in various habitual (e.g., Yin and Knowlton, 2006) and motivated (e.g., Ono et al., 2000) behaviors. It seems likely that these regions and their IC connections inform the processing of auditory stimulation and bias cells within the IC toward processing behaviorally important sounds. It is possible that the firing rate modulations found in these studies help to focus attention on a particular sound within the environment that will prove useful in, for example, detecting food or avoiding predators and other environmental dangers.

### Communication

Another form of specialized processing for which the IC appears to be particularly important is vocal communication. The role of the IC in this regard seems to lie somewhere between the basic auditory processing of lower brainstem regions and the more complex representation of communication calls found in cortical regions (Portfors and Sinex, 2005). Both auditory and non-auditory signals likely contribute to processing communication stimuli, and in this section, we also consider how somatosensory signals may be particularly important for audiomotor learning and maintenance of vocalizations.

Evidence implicating the IC in vocal communication comes from a variety of species. Specifically, some neurons respond better to conspecific calls than to either white noise and pure tones [around 75% of cells in sIC (82% in ICX, 72% in dorsal cortex) and 25% in ICC of cats (Aitkin et al., 1994)], or to time-reversed calls [approximately one-third of cells in guinea pig, all subdivisions (Suta et al., 2003); and in the rat ICC (Pincherli Castellanos et al., 2007)], while others fire selectively to particular conspecific calls (ICC of bats: Klug et al., 2002). Furthermore, some neurons in the ICX of squirrel monkeys are suppressed by self-generated calls despite responding to acoustically similar vocalizations from other monkeys and other sounds (Tammer et al., 2004). In humans, unilateral lesions to the IC have been reported to impair recognition of speech sounds when presented to the contralateral ear (Fischer et al., 1995; Champoux et al., 2007). These data suggest that one possible role of the IC in communication processing is generally identifying species-specific and self-generated vocalizations.

The presence of non-auditory signals in the IC may contribute to communication processing in this region. Numerous studies in animals have illustrated integration of visual and auditory components of communication at other levels of the auditory pathway (e.g., Ghazanfar et al., 2005; Romanski, 2007), and in humans, visual stimuli such as lip-motion can change the perception of speech sounds (e.g., the McGurk effect: McGurk and MacDonald, 1976)^4^. One patient with a circumscribed unilateral lesion to the right IC [the same reported in Champoux et al. (2007)] displayed a deficit in processing McGurk stimuli when visual stimuli were shown in the contralateral (left) visual hemifield (Champoux et al., 2006). Although this is only one patient, the results suggest that the IC may play a role in audiovisual speech processing.

In addition to a possible visual influence on the processing of speech and other vocal communication in the IC, somatosensation may be extremely important for learning and fine-tuning the IC's responses to vocal communication. The motor system is also presumed to be heavily involved in speech learning and production [for a review of the motor theory of speech learning, see Hickok et al. (2011); and Hickok (2012)]. Briefly, it is thought that an efference copy of the motor command sent to the effector muscles involved in speech is also sent to the auditory system. This allows for a comparison of the motor command and the resulting emitted sound, both during early development and during later maintenance of vocal performance. Indeed, proper somatosensory feedback appears important for maintaining consistent generation of speech sounds. In particular, numbing of the lingual nerve (a peripheral branch of the trigeminal cranial nerve) results in abnormal and inconsistent (across individuals) speech generation deficits for vowels (Niemi et al., 2002), diphthongs (Niemi et al., 2004), and sibilant /s/ sounds (Niemi et al., 2006). It has also been found that both deaf and normal-hearing people are sensitive to perturbations of jaw movements during speech regardless of auditory feedback (Nasir and Ostry, 2008). These data collectively indicate that somatosensory feedback from the articulators is necessary for maintaining proper speech production, and that perturbations to the system are corrected based on the same, non-auditory, feedback. Because the lingual branch of the TN is apparently important in this process, connections from the TN to the IC provide a likely candidate for a site of the audiomotor integration involved in speech learning and maintenance.

The convergence of auditory and motor feedback in the IC, and the resulting shaping of vocal communication responses and learning, may be mediated by projections from the periaquaductal gray matter (PAG) to the sIC (including ICX) (Dujardin and Jurgens, 2005). Previous studies have shown that vocalizations can be elicited through electrical stimulation of the PAG (rhesus monkey and cat: Magoun et al., 1937; squirrel monkey: Jurgens and Ploog, 1970; gibbon: Apfelbach, 1972; bat: Suga et al., 1973; guinea pig: Martin, 1976; rat: Yajima et al., 1976). Transections of the forebrain and SC preserve species-specific vocalizations in cats whereas transections caudal to the IC render these animals mute (Bazett and Penfield, 1922). Jurgens and Pratt (1979) investigated the role of the PAG in emotional expressions of squirrel monkeys with a series of lesions and stimulations. They found that lesions to the PAG disrupted induced vocalizations, and that motor output is likely accomplished via direct connections from the PAG to the nucleus ambiguus (NAm), the projection site for laryngeal motor neurons. One interesting possibility is that the PAG sends a corollary discharge to both the NAm and IC. This in turn may be used to cancel out the reafferent vocalization signal, which could explain why cells within the ICX do not respond to self-generated calls but still respond to calls from conspecifics (Tammer et al., 2004). Should this prove to be the case, there is evidence that the mechanism behind this process could be direct inhibition of the auditory system (after Suga and Shimozawa, 1974; Klug et al., 2002), or a more complex reafference-canceling signal that is susceptible to plastic changes according to the needs of the organism at that time (e.g., Bell, 1981).

**Figure 7:**
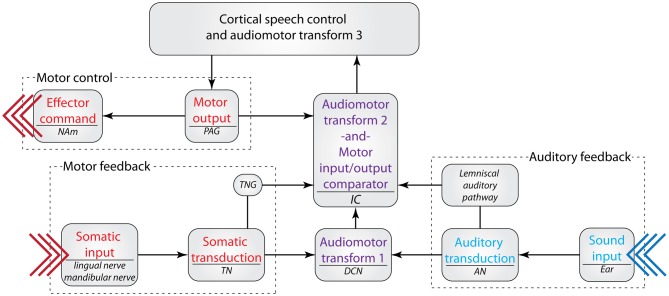
**A possible role for the inferior colliculus in audiomotor feedback during vocal communication.** The IC receives anatomical inputs from the PAG, TNG, DCN, and other auditory regions. This circuit, in theory, allows for a comparison between premotor commands sent from the PAG and somatic feedback from the vocal apparatus via the TN and TNG. The resulting comparison could yield a vocalization motor error: expected motor execution (output) minus actual motor execution (feedback) equals motor error. This information could be used to adjust the motor output of vocalization in order to achieve a desired auditory structure [presumably determined by cortical planning regions; see Hickok (2012)]. If the motor error = 0, self-generated vocalization reafference is cancelled out (Tammer et al., 2004) and no adjustment is necessary; if the motor error ≠ 0, the corresponding audiomotor error is sent to cortical control regions for further analysis and correction. The potential role of the DCN in this circuit is currently unknown, but may be associated with monitoring audiomotor feedback of non-vocalization signals. See Table 1 for abbreviations.

While few of these studies have focused on the IC to date, the prominent innervation of IC from somatosensory and behaviorally relevant sources suggests that it may be a crucial relay point in audiomotor feedback for vocal learning and maintenance of proper vocal production. It is possible that vocal motor commands that are sent from cortical control regions (e.g., the classic speech pathway) converge on the IC with auditory and somatosensory feedback. This potential circuit would allow for a direct comparison between the intended audiomotor output and the actual audiomotor execution at the level of the IC, which would in turn help to tune cortical audiomotor control mechanisms (Figure 7). This potential microcircuit has yet to be tested with respect to vocal communication. However, these pieces of circumstantial evidence suggest that non-auditory inputs to the IC may be involved in developing and maintaining vocal communication.

## Considerations for past results and future research

The presence of non-auditory signals throughout all subdivisions of the IC—including both ascending and descending regions—provides a point of entry for these signals to reach auditory processing at all stages from brainstem to cortex (note, for example, projections from the IC to both the thalamus and cochlear nucleus: Coomes and Schofield, 2004). In addition to having implications for our understanding of the role of the IC in auditory-guided behavior, as discussed in the preceding sections, there are several practical implications from a methodological perspective. Specifically, the presence of these signals suggests that non-auditory sensory stimuli and behavioral state must be included on the list of factors to be controlled, monitored, or randomized. This is true regardless of what level of the auditory pathway is under investigation, since signals present in the IC can be assumed to propagate to most, and probably all, areas of the auditory pathway.

In addition, certain surgical practices may affect the signals reaching the IC. For example, in order to more easily access the IC for electrophysiological recordings, the visual cortex is sometimes aspirated. Aspiration of the visual cortex is likely to alter the function of IC cells, and may do so in anywhere up to 80% of cells in the sIC and up to 25% of cells in the ICC [based on the proportions found by Bulkin and Groh (2012b)]. While these proportions assume the worst-case-scenario that all visual cells in the IC receive either direct or indirect influence from the visual cortex, clearly a substantial proportion of cells may undergo changes in their inputs after cortical aspiration. How this might affect auditory responses is uncertain.

Similarly, decerebration limits or destroys many of the described non-auditory and descending auditory inputs to the IC. While this method in particular has given (and continues to give) valuable insight into basic auditory processing, a complete understanding of more complex auditory processes, including sound localization and communication behaviors, will require that the brain be fully intact (Bazett and Penfield, 1922).

Pharmacological manipulations may also affect a substantial number of cells in the IC. Anesthetization of animal models has been shown to alter processing in the auditory cortex and thalamus (Zurita et al., 1994; Szalda and Burkard, 2005), IC (Kuwada et al., 1989; Szalda and Burkard, 2005), and CN (Evans and Nelson, 1973; Chen and Godfrey, 2000; Anderson and Young, 2004). With regard to non-auditory influences, anesthetization presumably has a substantial impact on the dynamic firing patterns of task-related neurons in the IC, a proportion consistently reported to be over half of the cells in this brain area. Induced paralysis, meanwhile, has the potential to influence the somatosensory signals in up to 75% of IC cells. In either case, pharmacological manipulations run the risk of inducing unintended alterations of auditory function, especially in the IC.

The above practices have given invaluable insight into numerous aspects of auditory processing. However, they may alter auditory function in unanticipated ways. Without explicitly testing the changes these methods may impart on the auditory system, there is no way to correct acquired data *post-hoc*. A thorough understanding of how the auditory system operates, particularly in its natural cognitive and broader sensory milieu, requires avoiding the unintended effects of structural and persistent pharmacological manipulations as much as possible. In particular, the use of awake and intact animals, when feasible, ensures that such pitfalls are circumvented.

Finally, we note that this review drew on the available evidence from a wide array of species, including mammals such as rodents, cats, and monkeys as well as birds such as barn owls. The demands on the sensory systems of different species may be different, and the neural organization and connections between sensory systems may differ accordingly. Further comparative work will ultimately be required to shed light on how the evolutionary history and ecological niche of different species are reflected in the patterns of non-auditory signals present in their ICs.

## Concluding remarks

The anatomical and physiological evidence that non-auditory factors contribute to IC activity is extensive. The roles played by the visual, oculomotor, eye position, somatosensory, and task-related signals are at present poorly understood, and may span a range of different aspects of auditory, multisensory, cognitive, and behavioral functions. Notable possibilities include integrating visual and auditory space, orienting to sounds, distinguishing self-generated from external sounds, accurately perceiving communication sounds, and monitoring vocal-related signals to achieve desired auditory performance. That such signals exist at an early, pre-cortical stage of the auditory pathway only a few synapses removed from sensory transduction in the cochlea highlights the importance of such constructive processes in the brain in interpreting sound.

### Conflict of interest statement

The authors declare that the research was conducted in the absence of any commercial or financial relationships that could be construed as a potential conflict of interest.

## Footnotes

^1^Though the subnuclear anatomy of the IC has been well described elsewhere (e.g., Morest and Oliver, 1984), the names and precise borders of these nuclei are not used consistently in the studies discussed in this review. Therefore, this core-shell nomenclature is adopted to avoid potentially applying these more precise subnuclear definitions inappropriately, particularly when such information is missing or ambiguous. When information about subnuclear locations is available for a given study, we have attempted to include the terminology used by the authors in our description of their findings.

^2^They estimated that approximately one-third of their sampled neurons were sensitive to audiovisual stimuli; however, the aim of their study was to describe the response patterns of these neurons, so they did not report an exact proportion.

^3^A possible role in communication behaviors will be discussed in the section on “Communication.”

^4^The McGurk effect (McGurk and MacDonald, 1976) is a common paradigm used to test audiovisual speech perception. In this and related studies, the auditory sound /ba/ is combined with a video of a person saying /ga/ (or some similar pair of related phonemes). Typically, an observer of the incongruent audiovisual pairing will perceive the intermediate phoneme /da/.
